# Our Hospitals in Atlanta

**Published:** 1887-08

**Authors:** T. S. Powell

**Affiliations:** Superintendent of Ivy Street Hospital


### OUR HOSPITALS IN ATLANTA.

### REPLY TO DR. GRAY'S STRICTURES UPON THE COUNCIL.

I hold that no man can be a friend to a community who will write and publish any views or opinions that he does not believe to be true, and calculated to honor and advance the best interests of the people among whom he lives, and from whom he receives his support. It is legitimate and proper for every man to advance his private interest in any honorable way, but he should not do so by presenting a false argument, nor should he vindicate wrong by upholding a friend whose course is unjust, and whose methods are not in accord with correct principles.

It is the duty of the press, and all classes of journalists, to stand as the sentinel upon the watch-tower to give warning of danger, and to protect and urge the right, and by no means endorse the wrong. The advocacy of wrong from ignorance, or from thoughtlessness, is a misfortune, but to do so for a consideration, as is so often done by attorneys, physicians, politicians, and by journalists, is a gigantic evil of the present day. While all such may be excused by themselves and society, they will, in the great day of account, in my judgment, find that compensation for a wrong done will give no exculpation for the doer.

I have penned the above in a general sense, and with no intention to wound or to injure any one, but in the hope that they may do good. I have meant them for all, and to every one to whom it may apply; but they were suggested, and, I feel are demanded of me, by an article which appeared in *The Journal*, June 30th, entitled "Some Salty Words," about how the city pays her hospital bills. It is stated that the article referred to is from the pen of Dr. James A. Gray, and one would infer from the caption given by the editor that he indorses the contents of the paper, which were plainly written to disparage the conduct, if not the honor, of our City Council, and to injure the usefulness or to destroy two of the hospitals in the city, to-wit: St Joseph's Infirmary and the Ivy Street Hospital. The writer reflects upon the justice and judgment of the Council for paying St. Joseph's Hospital $1 per day for each patient sent there by the city, and to the payment of 75 cents a day for the white patients sent to the Ivy Street Hospital, and the appropriation of $125 per month for all the negro patients which the Council may send to that institution. He makes the supposition that if only ten colored patients were sent in the course of the year that the cost would be extraxagant, but he is not posted in regard to the number that are monthly cared for and fed in the Ivy Street Hospital, which were shown last year in my report to the Relief Committee of Council to have cost the Council even less per capita than the boasted economy and luxuriant living that the writer claims for the Benevolent Home. Fearing that my language might deceive the Council as to the actual cost of feeding and the nursing the patients and running the entire concern, I stated in my report that while the cost to the Council did not reach by a fraction as much as that of the Benevolent Home, it had really cost the Ivy Street Hospital more than twice that amount, the deficiency being met by pay-patients, mostly from outside the city, and bv contributions of benevolent ladies in Atlanta.

It cannot be charged that the members of Council are wanting in judgment or economy, when with all the facts before them they know that the present cost of caring for the patients at the Ivy Street Hospital is 100 per cent. less than they could be treated and cared for in a hospital entirely under municipal control, for then there would be added the cost of medical treatment, now gratuitously furnished, the salaries of officers and nurses, and all the incidentals necessary to a large establishment of this kind. I infer that the writer of the article under review thinks that the Benevolent Home ought alone to be sustained by the Council by reason of its cheapness. I have shown that so far as the Council was concerned, the cost at the Ivy Street Hospital was even less, though to the hospital itself it was more. It should be borne in mind also that the medical treatment at the Benevolent Home is at the expense of the city, while it cost nothing at the Ivy Street Hospital, though the services be performed by older and more experienced physicians. While the preference for the Benevolent Home seems to be his thought, he advocates the establishment of a hospital by the city, to be under the separate and independent control of the Council. It would appear that the mind of Dr. Gray has, from some cause, recently undergone a change on this subject, as only a few months ago, when Dr. Ernest called a meeting of the physicians of the city to discuss the proposition of establishing an independent city hospital, the resolution was laid on the table, on the ground that there was already ample hospital accommodation for all the demands of the city. Dr. Ernest, the mover of the resolution, was the only man who voted for it, and Dr. Gray, who now favors such a project, we are informed, voted with the majority against it. I was not present at that meeting, but I published an article on the subject, giving my views, which were in full accord with Dr. Gray and every member at the meeting save one. I have often presented this view, and still hold that the time for a mammoth hospital in Atlanta has not arrived. The city would not erect a small or inferior building, and thanks to the extraordinary health of Atlanta, she does not need a large one. It were folly to add to our already heavy taxes the useless burden of not less than $100,000 for the erection of a large hospital, with paid physicians, nurses and salaried officers, when we have now more than sufficient hospital accommodation for all the demands of the city for many years to come, and at a cost of one-third, if not one-half, less than would be in an independent city hospital run wholly at the expense of the municipal authorities. Such an institution must incur the same expense for officers, whether empty or full of patients, and not being needed for our people, would become a sort of standing asylum for paupers, tramps and vagabonds, who would flock here for refuge and protection, greatly adding to our burdens for supporting this class of mendicants, which is heavy enough now in all reason. I opine that the tax payers of this city are not anxious to add to this class of incumbrances just yet. Certainly not in the face of the fact that their own wives and daughters have established an ample hospital, and have opened it to the city free of interest on investment, free of rent, and free of charge for medical attention, and have placee it in the hands of a "board of trustees of her best citizens."

At the time it was proposed there was no hospital in the city, the Benevolent Home being then used and intended as a home for old and destitute people, without special reference to medical treatment. The Ivy Street Hospital was established by the Ladies' Hospital Association, for hospital purpose only, wherein the really diseased and wounded persons could be cared for and scientifically treated. It is this class of patients only that are now provided for at the Ivy Street Hospital, and if the Benevolent Home had kept to its original design and confined itself to the class of people for whom it was inaugurated, there would now be no conflict of sympathy or interest and the Council could more easily and satisfactorily classify and distribute the destitute and afflicted. The Council and citizens generally would then know better how to appreciate the efforts of those who have labored so diligently and who have made so many sacrifices in establishing and running this institution. There is one fact that I will bring to the memory of the Council and the people—it is : that when the Ivy Street Hospital was first opened, the ladies of the Association made an arrangement with the President of the Southern Medical College, by which the Faculty of the College agreed to treat the city patients gratuitously. They deeded to the Trustees of the College the property to hold in trust for hospital purposes only, the Faculty having no pecuniary interest in the Hospital whatever, but to have the privilege of exhibiting to the medical class for the benefit of medical science, such patients as would in no way be offended or injured by such privile e. Herein lies the animus of the displeasure of those who imagine that this very just and reasonable privilege extended to the Faculty of the Southern Medical College, may possibly compete disadvantageously with some like enterprise in which they are engaged. I am sorry that anyone who claims to be a friend to Atlanta and to suffering humanity, and who desires the friendship and respect of the benevolent hearted ladies, who have aided in the establishment of, and are still laboring for this charitable enterprise, should be found to oppose this useful and important institution. Immediately after the establishment of the Hospital above referred to, the ladies addressed a polite and beautiful note to the city authorities, informing them of what they had done, and offering the city the benefits of the Hospital, with its free medical treatment. At that time the Council had no patients to send them, but at a subsequent meeting the gallant R. J. Lowry offered a beautiful resolution thanking the ladies for what they had done and their kind offer, and promising that when they needed hospital accommodations they would entertain their proposition. After this the Council made the proposition of a certain definite appropriation for white and colored patients, which has annually been renewed and accepted, and a moment's reflection will show to any intelligent man that what the Council pays the Ivy Street Hospital is not an exorbitant price for the accommodations furnished, but a positive saving and benefit to the city.

Endorsed and published by order of the Faculty of the Southern Medical College.

T. S. POWELL, M. D.,
Superintendent of Ivy Street Hospital.

